# Combination Therapy of VEGF-Trap and Gemcitabine Results in Improved Anti-Tumor Efficacy in a Mouse Lung Cancer Model

**DOI:** 10.1371/journal.pone.0068589

**Published:** 2013-07-09

**Authors:** Shuang Zhou, Yang Yang, Yaoqin Yang, Huihong Tao, Dong Li, Junli Zhang, Gening Jiang, Jianmin Fang

**Affiliations:** 1 Department of Histology and Embryology, Tongji University School of Medicine, Shanghai, China; 2 School of of Life Science and Technology, Tongji University, Shanghai, China; 3 Department of Thoracic Surgery, Shanghai Pulmonary Hospital, Tongji University School of Medicine, Shanghai, China; 4 Yantai Rongchang Biotechnologies, Ltd., Yantai, Shandong, China; Henry Ford Health System, United States of America

## Abstract

**Background:**

Angiogenesis is essential for the growth and metastasis of cancer. Although anti-angiogenic agents, particularly vascular endothelial growth factor (VEGF) inhibitors, have exhibited single-agent activity, there is considerable interest in combining these novel drugs with conventional chemotherapy reagents to achieve an optimal clinical efficacy. The objective of this study was to evaluate the benefits of the combination therapy of vascular endothelial growth factor trap (VEGF-Trap) with gemcitabine in a lung tumor model.

**Methods:**

A luciferase-expressing Lewis lung carcinoma (LLC) model was established in C57BL/6J mice and tumor-bearing mice were randomized into control, VEGF-Trap, gemcitabine and VEGF-Trap/gemcitabine combination groups. Tumor growth and animal survival were monitored. Tumor microvessel density and cell proliferation were evaluated by CD31 and Ki-67 immunohistochemical analysis. TUNEL assay was performed to detect apoptotic cells. The protein levels of Cyclin D1, Pro-Caspase-3, Bcl-2, MMP2 and MMP9 in tumor extracts were examined by western blot.

**Results:**

VEGF-Trap in combination with gemcitabine showed significantly enhanced inhibition of tumor growth and prolonged mouse survival compared to the VEGF-Trap or gemcitabine monotherapy. The VEGF-Trap/gemcitabine combination therapy not only potently inhibited tumor angiogenesis and cell proliferation, but also increased cellular apoptosis within tumor tissues. In addition, the combination treatment markedly down-regulated the expression of proliferation, anti-apoptosis and invasion related proteins.

**Conclusion:**

Combination therapy using VEGF-Trap and gemcitabine resulted in improved anti-tumor efficacy in a lung cancer model and VEGF-Trap/gemcitabine combination might represent a promising strategy in the treatment of human lung cancer.

## Introduction

Angiogenesis is the process of new blood vessel formation from existing vasculature. It is an important process in the development of malignant tumors as neoplastic lesions need to establish their own blood supply to grow beyond 1–2 mm in diameter [1]. The predominant regulator of tumor angiogenesis is vascular endothelial growth factor (VEGF) [2], [3], which is the key angiogenic factor known to be present throughout the entire tumor lifecycle [4]. Its continuous expression, along with the genetic stability of VEGF receptors in endothelial cells, makes direct and constant suppression of VEGF signal an important anti-tumor strategy [3], [4]. With the role of angiogenesis in tumor growth and progression firmly established, anti-angiogenic agents have received much widespread attention but clinical strategies for their optimal use are still being developed [5].

The anti-angiogenic agent vascular endothelial growth factor trap (VEGF-Trap or aflibercept) is an engineered chimeric protein containing the extracellular domain 2 of VEGF receptor-1 (VEGFR-1, Flt-1) and extracellular domain 3 of VEGFR-2 (KDR) fused to the Fc portion of human immunoglobulin G1. VEGF-Trap potently blocks all VEGF-A isoforms and PlGF of both human and mouse origins [6]. VEGF-Trap exerts its anti-angiogenic effects through regression of tumor vasculature [7], [8], normalization of surviving vasculature, and inhibition of new tumor vessel sprouting [9], [10]. These promising results led to the advancement of this agent in clinical studies [11], and it has been recently approved by U.S. FDA for colorectal cancer [12]. However, it is unlikely that anti-angiogenic therapies are curative on their own because anti-angiogenic agents themselves cannot kill tumor cells directly. Rather, their greatest potential may be realized when used in conjunction with conventional anti-cancer therapies, for example, chemotherapy [13], [14]. Anti-angiogenesis-induced normalization of tumor vasculature may improve the tumor perfusion and lead to enhanced chemotherapy delivery [15]–[18].

Lung cancer is the leading cause of cancer death worldwide. Gemcitabine is a deoxycytidine analogue that has shown efficacy as a treatment for many solid tumors. Gemcitabine has been commonly prescribed in the treatment of patients with non-small-cell lung cancer (NSCLC) [19], [20], however, its benefits are limited due to a low response rate or acquired tumor resistance.

In the present study, we attempted to evaluate the efficacy of the combination therapy using VEGF-Trap and gemcitabine in a mouse LLC lung cancer model, and to investigate the possible mechanism responsible for the increased anti-tumor efficacy.

## Materials and Methods

### Mice and Reagents

C57BL/6J female mice were purchased from the Chinese Academy of Science and housed at the Animal Maintenance Facility of Tongji University for at least 1 week prior to use. The protocols were approved by the Animal Ethics Committee of Tongji University. All animal experiments were performed under specific pathogen-free conditions in accordance with institutional guidelines. VEGF-Trap was constructed according to Holash and co-workers [8], and expressed in Chinese Hamster Ovary (CHO) cells after stable transfection. The recombinant VEGF-Trap was purified by protein-A affinity and ion exchange chromatography and reconstituted in sterile PBS. The quality of VEGF-Trap was determined by reducing SDS-PAGE. The binding activity of recombinant VEGF-Trap to mouse VEGF was confirmed by a ELISA-base direct binding assay. The protein was stored at −20°C until its use for subcutaneous (sc) administration at 1 g/kg. Gemcitabine (Gemzar) was kindly supplied by Eli Lilly (Indianapolis, Ind., USA) and stored at 4°C. According to the previous studies [21], gemcitabine was dissolved in sterile PBS for intraperitoneal (ip) injection at a dose of 60 mg/kg.

### Construction of Luciferase-expressing Tumor Cell Line

LLC mouse lung adenocarcinoma cells (CRL-1642) were from the American Type Culture Collection (ATCC), which were propagated in DMEM supplemented with 10% heat-inactivated fetal calf serum (GIBCO-BRL), 1 µM sodium pyruvate, 2 mM glutamine and 100 mg/l penicillin, and maintained at 37°C in humidified atmosphere containing 5% CO_2_ in air. Luciferase gene was amplified by polymerase chain reaction (PCR) from a cDNA template using a forward primer: 5′-CAC CGA CTC TAG AGC CGC CAC CAT GGA AGA TGC CAA AAA C and a reverse primer: 5′-CGG CAA GAT CGC CGT GTA ATA ACG GTC CGT CGA CAA TCA AC, flanked with X-bal I and Sal I restriction site. PCRs were performed with 30 cycles of denaturing at 95°C for 30 seconds, annealing at 58°C for 45 seconds, and extension at 72°C for 60 seconds. The PCR fragment was digested with X-bal I and Sal I and inserted into pRRL-CMV lenti-viral plasmid at X-bal I and Sal I site. Lenti-luciferase (lenti-Luc) viral vector was packaged in 293T cells by transfection of lenti-Luc and packaging plasmids (Invitrogen). After 48 hours post-transfection, the supernatants containing lenti-Luc virus were harvested. For lenti-Luc viral transduction, LLC cells were seeded in a 6-well plate and lenti-virus-containing supernatants were added to the medium (supernatants: fresh medium = 1∶1, approximately 1×10^8^ viral particles/ml) in the presence of polybrene (8 µg/ml). A stable luciferase-expressing clone was selected by limited dilution to create a Luc-LLC cell line. Luciferase activity in Luc-LLC cells was determined by luciferase assay using a luciferase detection kit (Beyotime) and bioluminescent imaging (Berthold). Further comparisons of cell growth, migration and tumor forming ability of LLC and Luc-LLC cells were conducted by MTT assay, wound healing and subcutaneous tumor model assessment, respectively.

### Animal Tumor Model and Treatment Groups

To inoculate tumors, 2×10^6^ Luc-LLC cells were resuspended in 200 µl of serum-free DMEM medium and subcutaneously injected into the right flank of 6 to 8 week-old C57BL/6J mice. One week after Luc-LLC inoculation, tumor-bearing mice were randomly divided into the following groups (n  = 8 per group) based on the bioluminescence measured after the first imaging and tumors volume reaching around 100 mm^3^: (a) untreated control (200 µl PBS thrice weekly by intraperitoneal injection); (b) VEGF-Trap alone (1 g/kg thrice weekly by subcutaneous injection); (c) Gemcitabine alone (60 mg/kg thrice weekly by intraperitoneal injection); (d) VEGF-Trap/Gemcitabine combination (VEGF-Trap 1g/kg thrice weekly by subcutaneous injection, and gemcitabine 60 mg/kg thrice weekly by intraperitoneal injection). Therapy was continued for 2 weeks thereafter. Tumor size was monitored every other day for 15 days with a vernier caliper after treatment initiation. Tumor volumes were determined using the formula: volume (mm^3^) = *a*×*b* ^2^×0.5, where *a* is the long diameter and *b* is the short diameter as previously described [22]. All of the data are represented as means ± SE. The mice were subjected to imaging on day 12 after the start of treatment. On day 15 after treatment initiation, mice in all groups were sacrificed. Tumors were weighted and photos were taken after dissection. Half of the tumor tissue was fixed in periodate-lysine-paraformaldehyde (PLP) and O.C.T (Sakura Finetek) embedded for immunohistochemistry. The other half was snap-frozen in liquid nitrogen and stored at −80°C. Survival curve analysis was done in an independent treatment group. Mouse death was determined using a pre-determined criterion.

### Bioluminescence Imaging *in vivo*

The bioluminescence imaging was performed using an animal imaging system (NightOWL LB 983 Molecular Imaging System, Berthold). The mice were injected with an intraperitoneal injection of 150 mg/kg D-luciferin potassium salt (Caliper Life Sciences) in 200 µl DPBS. After 5 minutes of luciferin injection, the mice were anesthetized via intraperitoneal injection of pentobarbital (50 mg/kg). Each mouse was placed in a left lateral decubitus position and a digital grayscale animal image was acquired, followed by the acquisition and overlay of a pseudocolor image representing the spatial distribution of detected photons emerging from active luciferase within the animal. The exposure time ranged from 10 to 30 seconds, depending on the intensity of bioluminescence emission from the tumor cells. Photons emitted from specific regions were quantified using an IndiGo software (Berthold). Regions of interest (ROI) were drawn around the tumor sites and quantified as photon counts per second.

### Immunohistochemistry

Tumor tissues were fixed in PLP, embedded in O.C.T, and cut into 10 µm sections. Then the sections were stained with specific antibodies for analysis.

For microvessel density analysis,tumor sections were stained with a rat anti-mouse CD31 antibody (BD Pharmingen) followed by FITC-conjugated rabbit anti-rat IgG (Invitrogen). Cellular nuclei were counterstained with DAPI (Sigma–Aldrich). The quantification of microvessel density (MVD) was assessed according to the method of Weidner et al. [23]. Briefly, the sections were firstly screened at low magnifications (×40 and ×100) to identify the most vascular area of the tumor (hot spot). Within the hot spot area, the stained microvessel was counted in a single high-power (×400) field. MVD was expressed as the number of microvessel/field. Any CD31-stained endothelial cells or endothelial cell clusters that were clearly separated from adjacent microvessels, tumor cells, or connective tissue elements were considered a single countable microvessel.

Immunohistochemical analysis of cell proliferation was performed on the frozen tumor sections with a rat anti-mouse Ki-67 antibody (Biolegend) followed by FITC-conjugated rabbit anti-rat IgG (Invitrogen). Results were expressed as the percentage of Ki-67 positive cells ± SEM per ×400 magnification. A total of ten ×400 fields were examined and counted from three tumors in each of the treatment groups. The Ki-67 proliferation index was calculated according to the following formula: the number of Ki-67 positive cells/the total cell count ×100%.

### TUNEL Assay

*In situ* detection of apoptotic cells in tumor tissue was performed by the terminal deoxynucleotidyl transferase–mediated dUTP nick end labeling (TUNEL) technique using a commercially available apoptosis detection kit (Beyotime) following the manufacturer’s protocol. TUNEL positive cells from 10 independent fields were quantified manually. The apoptosis index was calculated according to the following formula: the number of apoptotic cells/total number of nucleated cells ×100%.

### Western Blot Analysis

Tumor tissues on day 15 after treatment initiation were homogenized with lysis buffer (RIPA) (50 mM Tris–HCl, pH 7.4, 150 mM NaCl, 1 mM EDTA, 0.1% SDS, 1% Triton X-100, 1% sodium Deoxycholate, 1 mM PMSF, 10 mg/ml aprotinin, 10 mg/ml leupeptin) on ice. After centrifugation at 14,000 rpm at 4°C for 30 min, supernatants were collected and total protein concentrations were determined by BCA assay (Pierce). Equal amounts of denatured proteins were loaded onto 10% SDS-PAGE gel and transferred on PVDF membrane (Millipore). Membranes were blocked with 5% nonfat milk in TBST (1×TBS containing 0.1% Tween 20) and then incubated with anti-mouse primary antibodies to CyclinD1, Pro-Caspase-3, Bcl-2, MMP2 and MMP9 (all from Santa Cruz Biotechnology) overnight at 4°C. After washing with TBST three times, HRP-conjugated secondary antibodies were bound and performed with chemiluminescence using SuperSignal West Pico substrate (Pierce). Band intensities were quantified using Band Leader software.

### Statistical Analysis

Statistical significance was determined using one-way ANOVA or Student’s *t* test, as appropriate. ^*^ *P*<0.05 was considered statistically significant and ^**^ *P*<0.01 would be highly statistically significant.

## Results

### Establishment of LLC Tumor Model with Stable Luciferase Expression

**Figure 1:**
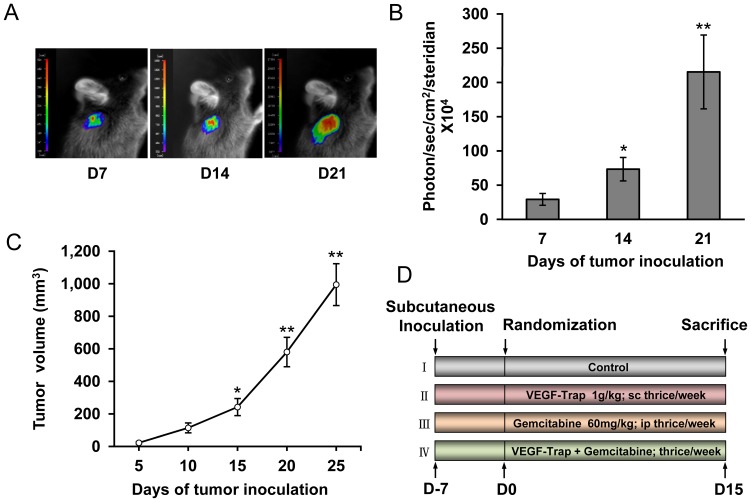
Establishment of LLC tumor model with luciferase stable expression. (**A**) Bioluminescence images of LLC tumors on day 7, 14 and 21 after Luc-LLC subcutaneous inoculation (n  = 6); (**B**) Measurements of photons per second depicting the tumor volumes of mice using the IndiGo imaging analysis software, ^*^ *P*<0.05, ^**^ *P*<0.01 *vs* day 7; (**C**) Tumor growth curve from day 5 to 25 after Luc-LLC subcutaneous inoculation (n  = 6), ^*^ *P*<0.05, ^**^ *P*<0.01 *vs* day 5; (**D**) Schematic representation of experiment protocol described in materials and methods, animals were divided into four groups: (a) Control; (b) VEGF-Trap alone; (c) Gemcitabine alone; (d) Combination of VEGF-Trap and Gemcitabine.

To better monitor tumor growth and metastasis *in vivo*, we constructed LLC cell lines with stable luciferase expression and subcutaneously inoculated Luc-LLC cells into C57BL/6J mice. Tumors were measured and imaged on day 7, 14 and 21 after tumor inoculation. As shown in Figure 1A and B, Luc-LLC tumor growth was visualized with bioluminescence imaging and photons emitted from specific regions were quantified using an IndiGo software, which was consistent with tumor growth curve (Figure 1C). No difference was seen between Luc-LLC and patent LLC cells in cell morphology, migration and growth *in vitro* as well as the tumor forming ability *in vivo* (Figure S2). Different treatment schedules were represented in Figure 1D.

### Effects of VEGF-Trap in Combination with Gemcitabine on LLC Tumors

**Figure 2:**
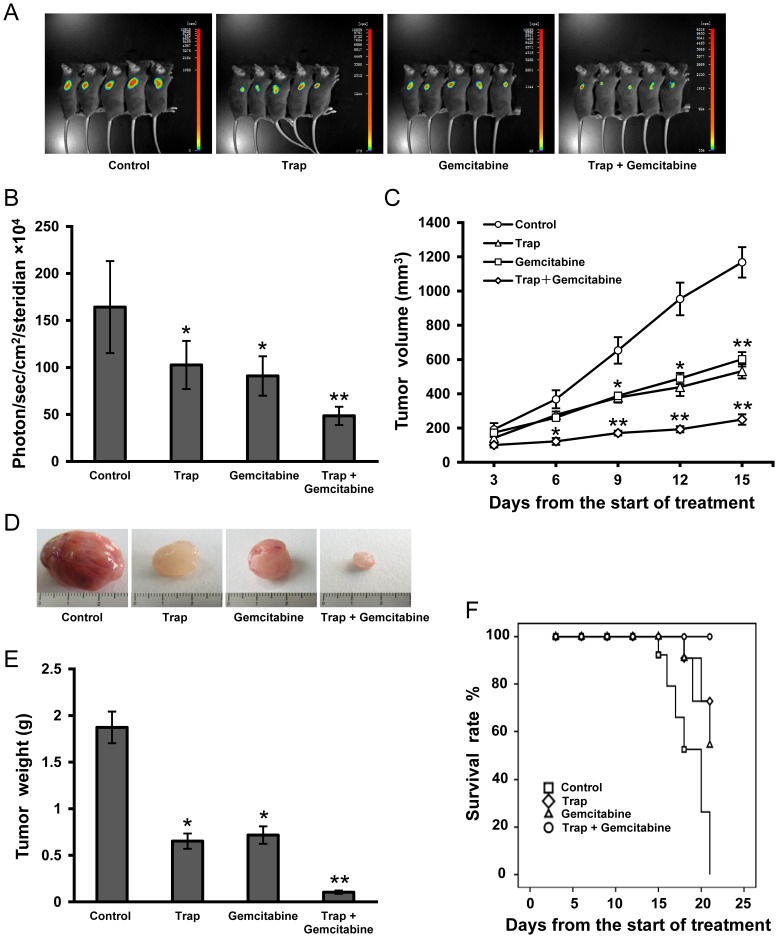
Anti-tumor effects of combination therapy with VEGF-Trap and gemcitabine in LLC tumor model. (**A**) Bioluminescence images of subcutaneous inoculated LLC tumors on day 12 after the start of treatment; (**B**) Imaging analysis (photons per second) depicting the tumor volumes of mice using the IndiGo imaging analysis software; (**C**) Tumor volume calculated on day 3, 6, 9, 12,15 after the start of treatment; (**D**) Representative tumor photos on day 15 after the start of treatment; (**E**) Tumor weights were measured on day 15 when tumors were harvested; (**F**) Survival curves were constructed according to the Kaplan-Meier analysis. n  = 8 for each group, *P<0.05, **P<0.01 *vs* the control group.

To investigate the therapeutic effects of combination therapy of VEGF-Trap and gemcitabine on the growth of LLC tumors, C57BL/6 mice bearing LLC tumors were divided into four treatment groups as described above. VEGF-Trap was prepared as described in the Materials and Methods. The purified VEGF-Trap showed as a single band with an apparent molecular weight of 50 kD (Figure S1A) and exhibited a potent binding activity to mouse VEGF as determined by a direct binding assay (Figure S1B). When the volumes of the tumors reached around 100 mm^3^, the treatment was initiated. On day 6 after initial treatment, VEGF-Trap and gemcitabine combination therapy showed significant inhibitory effects on tumor growth as compared to the control group. Although VEGF-Trap or gemcitabine alone also significantly inhibited tumor growth, the combination therapy resulted in a more robust inhibition on tumor development and had the most significant delay in tumor growth as determined by tumor volume on day 9, 12 and 15 after initial treatment (Figure 2C, *P*<0.01, compared with the control group). Bioluminescence imaging analysis of LLC tumors on day 12 after the start of treatment confirmed the anti-tumor effect (Figure 2A and B). Significant differences in tumor sizes and weight were seen on day 15 when treatment was stopped and tumors were dissected (Figure 2D and E). No statistically significant difference in tumor size was found between VEGF-Trap and gemcitabine monotherapy groups. Thus, the combination therapy of VEGF-Trap and gemcitabine increased anti-tumor efficacy in LLC tumor model. Similarly, the increased inhibition of tumor growth in the combination therapy was translated into prolonged animal survival according to the Kaplan-Meier analysis and the mice receiving the combination therapy remained at a 100% survival rate by the end of the observation period (Figure 2F).

### Inhibition of Tumor Angiogenesis and Cell Proliferation

**Figure 3:**
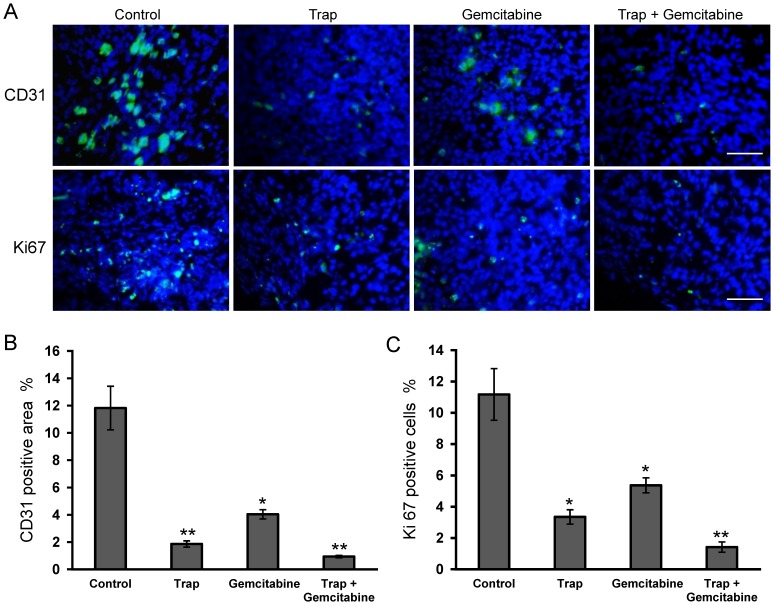
Inhibition of angiogenesis and cell proliferation within tumors after the combination therapy. (**A**) Representative images of CD31-positive microvessel area and Ki67-positive cells in the viable LLC tumor tissues on day 15 after treatment initiation were estimated by immunohistochemical staining; (**B**) and (**C**) Microvessel density and percentage of Ki67-positive cells were determined by counting the number of the positive staining per high-power field in the section, as described in “Materials and Methods”. *P<0.05, **P<0.01 *vs* the control group. Scale bar, 50 µm.

LLC tumors from all treatment groups were harvested on day 15 after the start of treatment and were prepared for immunehistochemistry studies as described in Materials and Methods. Angiogenesis within tumor tissue was evaluated by counting microvessel density following immunohistochemical staining for CD31. Treatment with VEGF-Trap or the combination therapy resulted in an obvious inhibition of the angiogenesis in tumors compared to the control group. The average vessel counts per high-power field in VEGF-Trap or combination group were significantly lower (*P*<0.01) than that in the control group (Figure 3A and B). Although there was a statistical significance in microvessel density, we also noted that gemcitabine-treated tumors showed a stronger CD31-positive area than the tumors in the combination therapy group.

To examine cell proliferation in tumor tissues after treatment, frozen tumor sections were stained with Ki-67 antibody and the Ki-67 proliferation index was calculated based on the immunohistochemical analysis. The Ki-67 proliferation index showed an 8-fold decrease in VEGF-Trap and gemcitabine combination group compared to the control group (Figure 3A and C), suggesting a significant suppression of tumor cell proliferation in the treatment groups.

### Increase in Tumor Cell Apoptosis

**Figure 4:**
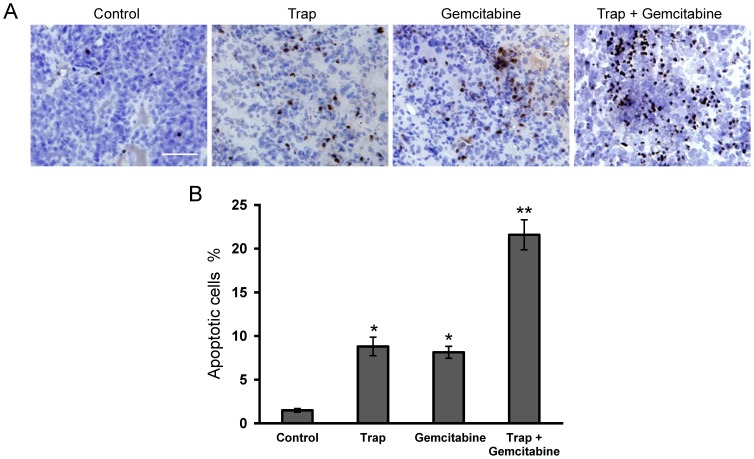
Effect of VEGF-Trap combined with gemcitabine on cell apoptosis within tumors. (**A**) Cell apoptosis was evaluated by TUNEL staining, the representative sections of LLC tumor tissues were obtained from mice on day 15 after treatment initiation; (**B**) The apoptotic index was determined as described in “Materials and methods”. *P<0.05, **P<0.01 *vs* the control group. Scale bar, 50 µm.

To further investigate the mechanism of the combined effects of VEGF-Trap and gemcitabine on LLC tumors, a TUNEL assay was performed to study tumor cell apoptosis. As shown in Figure 4A and B, the TUNEL assay revealed a marked increase in apoptosis within tumor tissues from the combination therapy group (*P*<0.01), when compared with the other groups.

### Down-regulation Expression of Proliferation, Anti-apoptosis and Invasion Related Proteins

**Figure 5:**
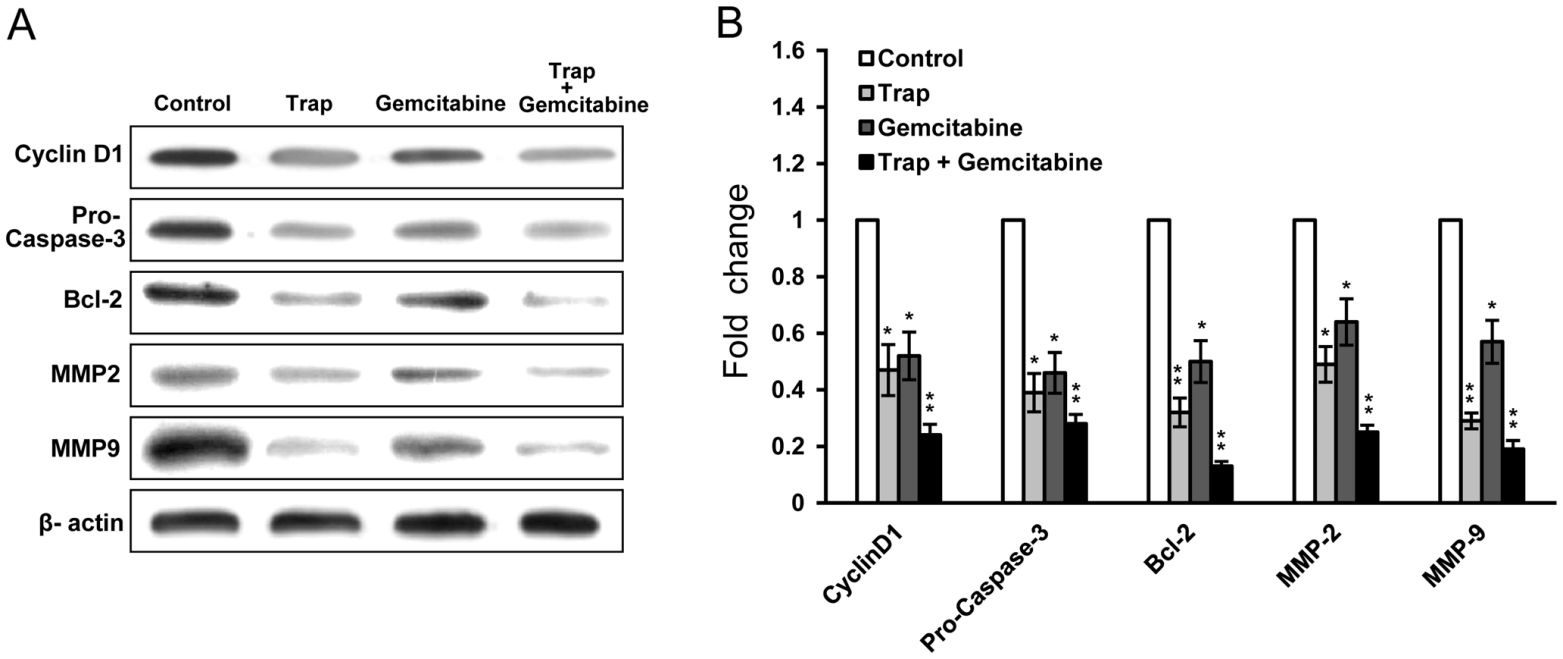
Down-regulation of proteins associated with proliferation, anti-apoptosis and invasion in tumor after the combination therapy. (**A**) Western blot analysis showed that VEGF-Trap combined with gemcitabine inhibited the expression of Cyclin D1, Pro-Caspase-3, Bcl-2, MMP2 and MMP9 in LLC tumors; (**B**) Quantitation of protein levels. β-actin served as an internal control. Densitometer quantitation was relative to the first data set in each case (indicated by a value of 1). All data are representative of at least two independent experiments. *P<0.05, **P<0.01 *vs* the control group.

Having established the changes in microvessel density, cell proliferation and apoptosis index in response to VEGF-Trap and gemcitabine combination therapy, we next sought to investigate the possible mechanism of the combined effects observed in the tumor model *in vivo* by assessing the expression of cell proliferation (Cyclin D1), anti-apoptosis (Pro-Caspase-3, Bcl-2), and invasion (MMP2, MMP9) related proteins using western blot. In the extracts from LLC tumors on day 15 after the start of treatment, Cyclin D1, Pro-Caspase, Bcl-2, MMP2 and MMP9 expression were down-regulated in VEGF-Trap and gemcitabine combination group in comparison with the control group (*P*<0.01). Western blot results confirmed that VEGF-Trap or gemcitabine monotherapy could down-regulate the expression of all these proteins, but combination therapy further enhanced these effects (Figure 5A and B). This data was consistent with the results from immunohistochemical analysis and TUNEL assay (Figure 3, Figure 4).

## Discussion

Angiogenesis is a tightly regulated process, involving a dynamic balance between stimulatory and inhibitory signals. Angiogenesis occurs when the balance between angiogenic stimulators and inhibitors is in favor of stimulators [24]. The blockade of the key angiogenic stimulator, VEGF, is an effective means to inhibit angiogenesis in animal tumor models [25], [26]. However, anti-VEGF monotherapy has limited clinical benefits for cancer patients and in high dosage can often lead to various adverse effects such as hypertension, proteinuria, thromboses, and GIP [27], [28]. Therefore, further research is required to maximize the anti-tumor effect while minimizing the number of side effects from anti-angiogenic therapy.

Increasing evidence has suggested that the combination of angiogenesis inhibitors with chemotherapy produced better therapeutic outcomes in the treatment of cancer [29]–[35]. A phase III clinical trial demonstrated that a combination of anti-VEGF monoclonal antibody bevacizumab with carboplatin–paclitaxel significantly improved overall survival (OS) in lung cancer patients compared to chemotherapy alone [36]. Thus, it is clinically relevant to explore more combinations between anti-angiogenic agents and chemo regimens for more therapeutic options.

Given the wide use of gemcitabine in the treatment of NSCLC, it is important to determine if the combination of an anti-angiogenic agent with gemcitabine can produce any clinical benefit. Recently, a phase III study (AVAiL trial) that compared bevacizumab plus cisplatin-gemcitabine to chemocherapy (cisplatin-gemcitabine) showed an improved progress-free survival, but not overall survival [37]. It remains a challenge to extend overall patient survival on top of gemcitabine therapy.

VEGF-Trap is a chimeric VEGF decoy receptor-Fc fusion protein and has been recently approved by U.S. FDA for metastatic colorectal cancer [12]. Compared to bevacizumab, VEGF-Trap is a more potent angiogenesis inhibitor due to its higher affinity to VEGF. In addition, VEGF-Trap can also block PlGF which is also a pro-angiogenic factor. Thus, it would be interesting to evaluate if the combination of VEGF-Trap and gemcitabine can result in a more effective therapy for lung cancer.

In the present study, we hypothesized that a combination therapy comprised of VEGF-Trap and gemcitabine could achieve improved anti-tumor effects. To test this hypothesis, we examined the therapeutic effects of this combination therapy in a LLC lung cancer model. The results showed that combination treatment with VEGF-Trap and gemcitabine provided more therapeutic benefits than each individual modality. Combination therapy had significantly higher inhibitory effects on tumor growth and a prolonged survival rate, not only inhibiting tumor angiogenesis and cell proliferation, but also increasing cell apoptosis within tumor tissues.

To explore the possible mechanism underlying the improved anti-tumor efficacy in the VEGF-Trap and gemcitabine combination therapy, tumor tissue extracts were studied by western blot for protein expression. The results showed that VEGF-Trap or gemcitabine alone down-regulated the expression of proliferation (Cyclin D1), anti-apoptosis (Pro-Caspase-3, Bcl-2), and invasion (MMP2, MMP9) related proteins, but the combination therapy resulted in a more significant down-regulation of these markers compared to the monotherapy groups. This data was consistent with the results from immunohistochemical analysis and TUNEL assay, supporting the idea that combination therapy of VEGF-Trap and gemcitabine can improve anti-tumor efficacy.

The high potency of VEGF inhibition by VEGF-Trap makes this agent an ideal partner for combination with approaches aimed at other mechanisms of tumor progression. In clinics, VEGF-Trap therapy may offer additional benefits through normalization of tumor vasculature, which reduces interstitial pressure and vessel permeability, and increases the delivery of other agents into the tumor tissues, which may make tumor cells more sensitive to chemotherapy [38]–[40]. The combination of continuous VEGF inhibition by VEGF-Trap with other treatment modalities may represent a more optimal therapeutic option in a number of tumor types.

Our results suggested that the combination of VEGF-Trap and gemcitabine might be a more effective alternative for human lung cancer, which may form the basis of a rationale for human clinical studies to investigate the benefits of the combination therapy of VEGF-Trap and gemicitabine in lung cancer.

In conclusion, combination therapy of VEGF-Trap and gemcitabine resulted in improved anti-tumor efficacy in the LLC tumor model. The combination therapy inhibited tumor growth and prolonged animal survival through suppressed tumor angiogenesis and cell proliferation, as well as increased tumor cell apoptosis. VEGF-Trap/gemcitabine combination therapy might present a promising strategy for human lung cancer.

## Supporting Information

Figure S1 **The quality and activity of VEGF-Trap.** (**A**) The quality of VEGF-Trap was determined by reducing SDS-PAGE to show as a single band with an apparent molecular weight of 50 kD; (**B**) VEGF-Trap exhibited a potent binding activity to mouse VEGF as confirmed by a direct binding assay.
(TIFF)

Figure S2 **Comparison of biologic features of LLC and Luc-LLC cells.** Cell morphology and migration (**A**), growth (**B**), and tumor forming ability (**C**) of LLC and Luc-LLC cells were conducted by wound healing, MTT assay and subcutaneous tumor model assessment, respectively, which showed no difference between the transgenic and non-transgenic cells. Scale bar, 200 µm.
(TIFF)
