# Supplementary figures and images for: Combination Therapy of VEGF-Trap and Gemcitabine Results in Improved Anti-Tumor Efficacy in a Mouse Lung Cancer Model
Source: PLoS One. 2013 Jul 9;8(7):e68589. doi: 10.1371/journal.pone.0068589 (PMC3706404; doi:10.1371/journal.pone.0068589)

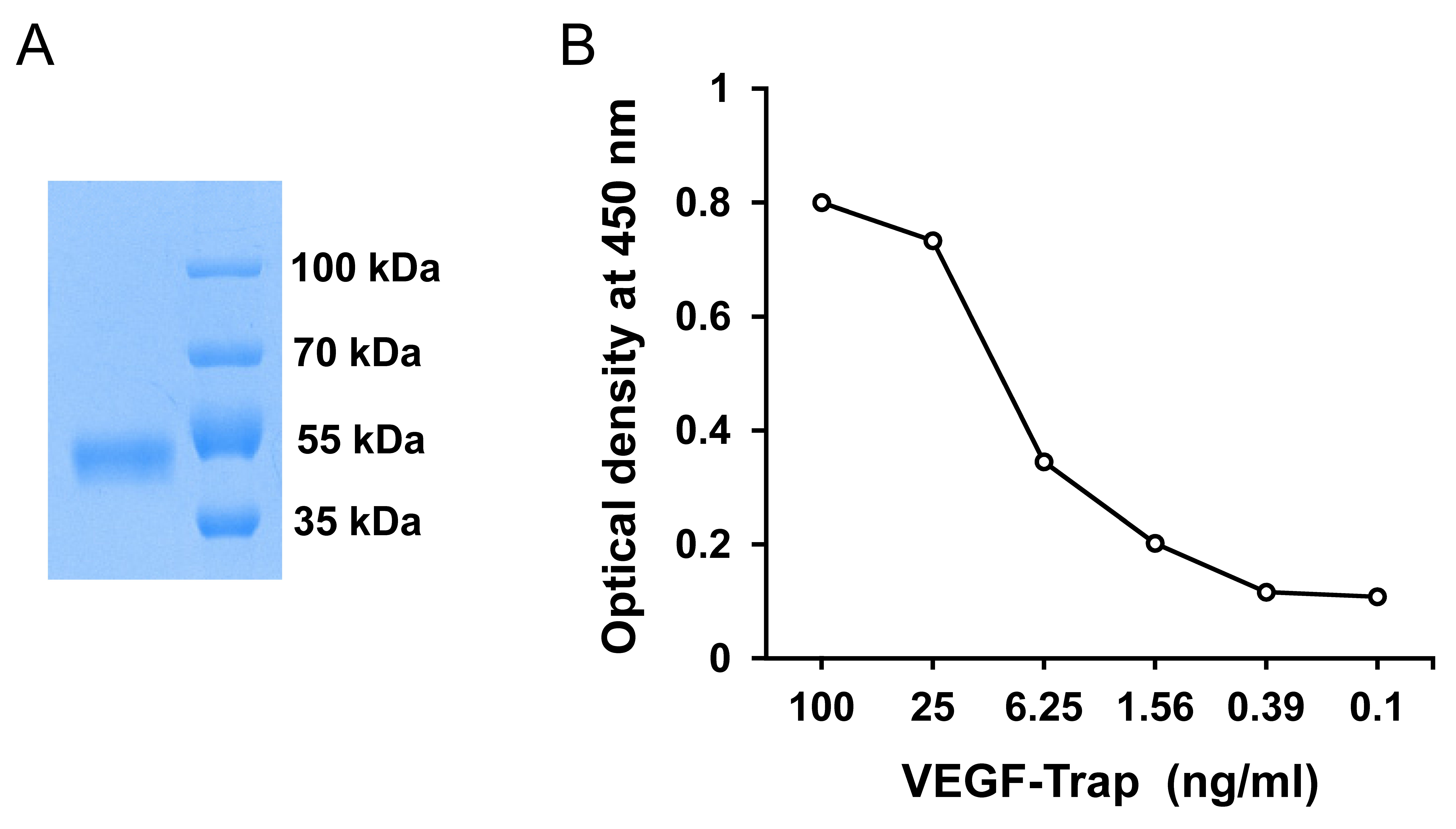

Supplement: Figure S1 — The quality and activity of VEGF-Trap. (A) The quality of VEGF-Trap was determined by reducing SDS-PAGE to show as a single band with an apparent molecular weight of 50 kD; (B) VEGF-Trap exhibited a potent binding activity to mouse VEGF as confirmed by a direct binding assay. (TIFF) [file pone.0068589.s001.tiff]

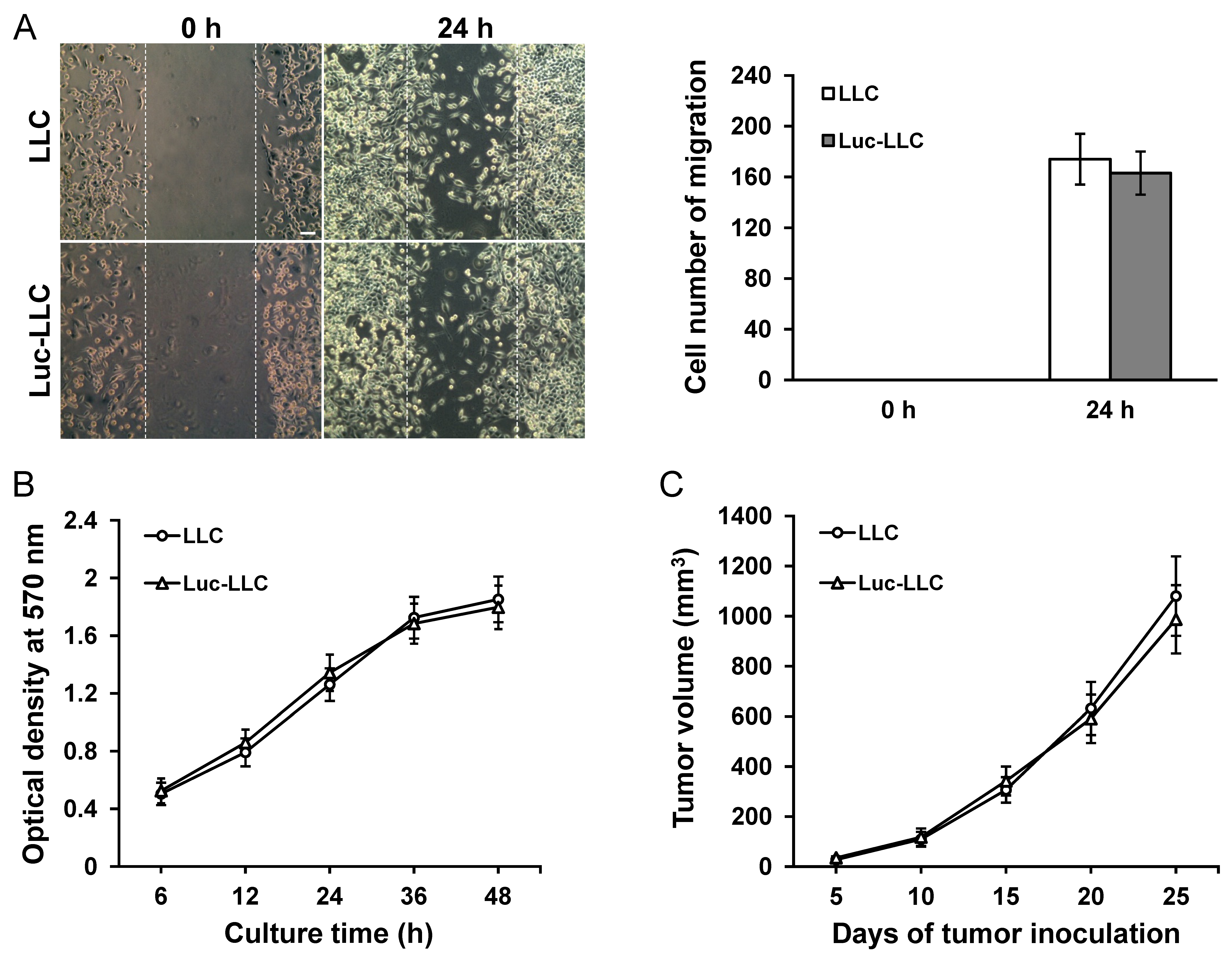

Supplement: Figure S2 — Comparison of biologic features of LLC and Luc-LLC cells. Cell morphology and migration (A), growth (B), and tumor forming ability (C) of LLC and Luc-LLC cells were conducted by wound healing, MTT assay and subcutaneous tumor model assessment, respectively, which showed no difference between the transgenic and non-transgenic cells. Scale bar, 200 µm. (TIFF) [file pone.0068589.s002.tiff]
